# Emotional modulation of cortical activity during gum chewing: A functional near-infrared spectroscopy study

**DOI:** 10.3389/fnins.2022.964351

**Published:** 2022-11-29

**Authors:** Yoko Hasegawa, Ayumi Sakuramoto, Tatsuya Suzuki, Joe Sakagami, Masako Shiramizu, Yoshihisa Tachibana, Hiromitsu Kishimoto, Yumie Ono, Takahiro Ono

**Affiliations:** ^1^Division of Comprehensive Prosthodontics, Faculty of Dentistry & Graduate School of Medical and Dental Sciences, Niigata University, Niigata, Japan; ^2^Department of Dentistry and Oral Surgery, School of Medicine, Hyogo Medical University, Nishinomiya, Japan; ^3^Department of Electronics and Bioinformatics, School of Science and Technology, Meiji University, Kawasaki, Japan; ^4^Electrical Engineering Program, Graduate School of Science and Technology, Meiji University, Kawasaki, Japan; ^5^Sakagami Dental Clinic, Nara, Japan; ^6^Department of Dental Hygiene, Otemae Junior College, Nishinomiya, Japan; ^7^Division of System Neuroscience, Kobe University Graduate School of Medicine, Kobe, Japan

**Keywords:** taste, odor, hemodynamic response, emotion, fNIRS, cerebral blood flow, chew

## Abstract

Distinct brain regions are known to be associated with various emotional states. Cortical activity may be modulated by emotional states that are triggered by flavors during food intake. We examined cortical activity during chewing with different flavors and assessed the emotional modulation of cortical activity using multichannel near-infrared spectroscopy. Thirty-six right-handed volunteers participated in this crossover trial. The participants experienced positive and negative emotions from chewing flavorful (palatable) or less flavorful (unpalatable) gums, respectively for 5 min. Participants rated the taste, odor, and deliciousness of each gum using a visual analog scale. Bilateral hemodynamic responses in the frontal and parietal lobes, bilateral masseter muscle activation, and heart rate were measured during gum chewing. Changes in all measured data during gum chewing were also evaluated. The ratings of the tastes and odors of each gum significantly differed among the participants (*P* < 0.001). Hemodynamic response changes were significantly elevated in the bilateral primary sensorimotor cortex during gum-chewing, in comparison to resting. The difference in hemodynamic responses between palatable and unpalatable gum conditions was detected in the left frontopolar/dorsolateral prefrontal cortex. Muscle activation and heart rate were not significantly different between different gum types. Our findings indicate that differential processing in the left prefrontal cortex might be responsible for the emotional states caused by palatable and unpalatable foods.

## Introduction

Chewing is necessary for digestion and absorption of various nutrients. In addition to these functions, chewing is tightly linked to the emotional centers of the brain (Deiss et al., 2009). Eating behavior depends on the self-perception of the flavor of food involving the sense of taste, smell, temperature, mechanoreception, and masticatory movement. Sensory signals are individually processed in the gustatory and olfactory neural circuits of the brain and then integrated as a sensation of taste in the insular and orbitofrontal cortical areas (Kringelbach, 2005; Shepherd, 2006). Information on flavors from the cortical areas is further transmitted to the brain reward system, including the nucleus accumbens, midbrain dopamine areas, amygdala, and hypothalamus (Shepherd, 2006; Haber and Knutson, 2010). Hedonic (pleasure–displeasure) responses represent the first level of emotional experiences (Hoffman, 1986). Emotional modulation of the hypothalamus affects the autonomic and motor functions of the body. Moreover, delicious meals relieve muscle tension and relax the mind and the body (Nakamura, 2005).

We previously showed that prefrontal cerebral blood flow during palatable gum chewing was higher than during unpalatable gum chewing (Hasegawa et al., 2013). The food-related stimuli, including flavor and taste, caused consistent activation of the prefrontal cortex, suggesting its active involvement in the emotional change during eating (Hasegawa et al., 2013). Stress causing changes in blood circulation and endocrine system is also known to alter eating behavior and contribute to the persistence of unpleasant emotions (Levine and Morley, 1981). We showed that the emotional changes due to taste and odor influenced the hemodynamic responses and the endocrine system functions (Hasegawa et al., 2017). Negative emotions reduce prefrontal cortex activity (Kim and Hamann, 2007). Previous research has shown that emotional changes are closely related to eating behavior, and pleasant/unpleasant memory is also strongly linked to eating behavior (Hernández Ruiz de Eguilaz et al., 2018). However, previous studies failed to compare the cortical activation between pleasant and unpleasant emotions during chewing.

Functional near-infrared spectroscopy (fNIRS) uses surface-mounted optodes to detect changes in the spectral absorbance of both oxyhemoglobin (oxy-Hb) and deoxyhemoglobin (deoxy-Hb) (Wyatt et al., 1990; Villringer and Chance, 1997; Strangman et al., 2002; Cui et al., 2011; Scholkmann et al., 2014). The principle of operation of fNIRS involves hemodynamic signals that serve as a proxy for neural activity, similar to hemodynamic signals acquired by functional magnetic resonance imaging (f-MRI) (Ferrari and Quaresima, 2012; Boas et al., 2014; Noah et al., 2015). For the hemodynamic response measured by fNIRS, the signal source is registered to standard brain coordinates, as in f-MRI. The fNIRS can also quantitatively evaluate neural activity with high temporal resolution on a channel set-up. This device is compact and exhibits no restrictions with respect to the measurement location. As the constraints during measurement are small, the fNIRS enables experiments in a real-world setting with limited body movement as during chewing (Okamoto et al., 2011; Tachibana et al., 2019; Nagashima et al., 2020). This method can omit head movements during the analysis. Therefore, the fNIRS is suitable for measuring brain activity during chewing.

Thus, this crossover study aimed to clarify whether cortical activity is related by emotional states triggered by chewing gums of different flavors. In particular, we focused on whether changes in the activity of the prefrontal cortex were affected by the emotions (palatable-unpalatable) during eating experience. We used the fNIRS to elucidate the cortical activity during palatable or unpalatable gum chewing related to positive/negative emotions.

## Materials and methods

The study protocol was approved by the Ethics Committee of the Hyogo College of Medicine (approve num-2209). The study protocol was registered with the UMIN CTR Japan Primary Registries Network (UMIN00025567). This study was conducted in compliance with the Declaration of Helsinki and the ethical guidelines for medical and health research involving human subjects established by the Ministry of Health, Labor, and Welfare in Japan. Written informed consent was obtained from all participants.

### Study protocol and sensory tests

Thirty-six right-handed volunteers (19 males and 17 females; mean age, 28.0 ± 4.0 years) without any medical/psychiatric disorder or medication use participated in this study. All participants provided written informed consent after receiving an explanation of the approved experimental protocol from the institutional ethics committee at Hyogo College of Medicine.

**FIGURE 1:**
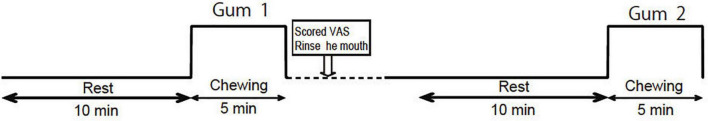
Experimental protocol. The order of the palatable gum and unpalatable gum (corresponding to Gum 1 or Gum 2) was random.

The experimental protocol is illustrated in Figure 1. All experiments were performed in a shielded room at 25°C. Chewing tests were performed at least 4 h after meals. The participants were instructed to sit on a chair with their necks supported by a headrest and their eyes gently closed.

We prepared two types of gums of different flavors: (Hasegawa et al., 2013), a lemon-flavored sweet gum (Free zone; Lotte, Tokyo, Japan) that could induce a positive emotion (palatable gum), and a salty licorice-flavored sweet gum (Lotte, Tokyo, Japan) that could induce a negative emotion (unpalatable gum). Salty licorice has a displeasing and unfamiliar flavor for most Japanese people from their first experience. The participants were instructed to chew the tested gum at a constant rhythm of 70 chews per minute using a metronome sound (Hasegawa et al., 2009). The gum was placed in the mouth of the participants immediately before the start of chewing and was removed by the examiner immediately after the end of chewing. The participants rated the taste/odor/deliciousness of each gum using a visual analog scale (VAS: 0–100 = Very bad-Very good) after completion. They rinsed their mouths with mineral water and rested for 10 min before chewing the next gum. To evaluate the association between the change in hemodynamic response and emotion, the relative value of the VAS was calculated using the following formula:


Palatable gum=X/{(X+Y)/2}, Unpalatable gum=Y/{(X+Y)/2}

X: VAS of palatable gum ⋅⋅

Y: VAS of unpalatable gum

### Near-infrared spectroscopy monitoring and data analysis

**FIGURE 2:**
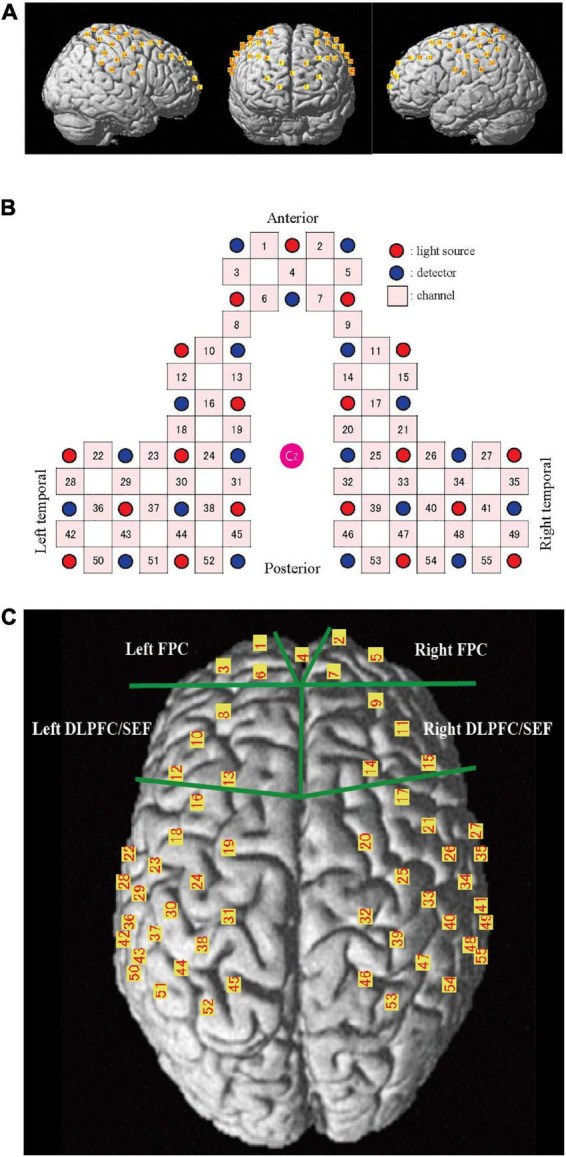
Channel location on the cortical surface. **(A)** Predicted location of each channel on the cortical surface. The front, right, and left hemisphere of a single-rendered brain illustrate the average locations of the 55 channels identified by number. **(B)** Schematic illustration of the location of each optode and channel where Cz represents the vertex. **(C)** Near-infrared spectroscopy (NIRS) channels and regions of interest (ROI). FPC, frontal pole; DLPFC/SEF, dorsolateral prefrontal cortex and includes frontal eye fields.

A 55-channel (ch) fNIRS system was used to detect the chewing-induced hemodynamic changes in the cerebral cortex (Figure 2). The fNIRS system used three wavelengths (780, 805, and 830 nm) of continuous near-infrared light (LABNIRS; Shimadzu Corp., Kyoto, Japan) with 19 light sources and 19 detectors and a sampling rate of 3.3 Hz. Each optode (light sources/detectors) was attached to the head surface using a custom-made hard plastic cap with an inter-optode distance of 3.0 cm. We defined the fNIRS channel as the midpoint of the corresponding light source-detector pair. The optodes were carefully arranged to avoid the temporal muscles that potentially cause the systemic and motion artifact by chewing behavior (Nakajima et al., 2020). The subjects were asked to perform tooth tapping several times before the measurement to confirm that the NIRS signal was not affected by temporal muscle activity.

The NIRS_SPM_v4 running on MATLAB (MathWorks, Natick, MA, USA) was used to position the probe and channels in the standard brain coordinates, obtained using a 3D digitizer, onto the cerebral cortex to assess the blood flow change. The position of the 55 channels was calculated as the midpoint between the emitter and the detection probes. Because the probe positions were available from six participants, we calculated the mean montreal numerological institute (MNI) coordinates for each channel and used them as the channel position coordinates of all participants. The cortical regions and Brodmann area (BA) corresponding to each of the 55 channels were estimated using the Anatomy 1.8 toolbox for SPM (Eickhoff et al., 2005; Table 1).

**TABLE 1:** Comparison of the hemodynamic responses during the chewing of palatable gum and unpalatable gum and comparison of the area under the curves (AUCs) during gum chewing.

	Unpalatable gum		Palatable gum		Palatable vs. unpalatable		
			
Channel	Mean	S.D.	Mean	S.D.	*P-value*	Cortical region	Brodmann area
CH1	−38.3	7454.1	−1442.3	5559.3	0.064	FPC	10
CH2	−716.7	8969.8	−605.6	4892.3	0.870	FPC	10
CH3*	−92.8	3432.8	−1244.0	2954.1	0.026	FPC	10
CH4	−627.8	3816.6	−617.1	2981.6	0.857	FPC	10
CH5	264.3	3678.4	−506.4	2883.6	0.185	FPC	10
CH6	38.5	1417	−581.7	1891.8	0.095	FPC, DLPFC	9, 10
CH7	−273.2	2294.9	−707.8	3635.4	0.682	FPC, DLPFC	9, 10
CH8	503.0	1128.8	−8.2	922.3	0.074	DLPFC, SEF	8, 9
CH9	−19.5	3181.2	−400.9	1996.8	0.922	DLPFC, SEF	8, 9
CH10	634.7	3117.4	238.6	1947.7	0.351	DLPFC, SEF	8, 9
CH11	42.4	3550.5	−400.7	1942.8	0.844	DLPFC, SEF	8, 9
CH12	271.3	1849.1	152.1	725.8	0.600	DLPFC, SEF	8, 9
CH13	219.0	658	445.0	921.3	0.258	SEF, PM/SMA	6, 8
CH14	223.1	1592.9	−195.8	1023.1	0.116	SEF, PM/SMA	6, 8
CH15	358.6	2610.8	224.3	1757.1	0.743	DLPFC, SEF	8, 9
CH16	205.9	1584.4	351.3	1114.5	0.883	SEF, PM/SMA	6, 8
CH17	386.9	1894.2	−35.2	1303.5	0.359	SEF, PM/SMA	6, 8
CH18	213.2	416.2	202.1	520.4	0.896	PM/SMA	6
CH19	171.8	483.4	250.5	535.5	0.566	PM/SMA	6
CH20	355.0	896.7	245.6	448.1	0.781	PM/SMA	6
CH21	516.6	866.7	155.8	1247.1	0.318	PM/SMA	6
CH22	1420.6	2243.2	1487.6	2280.6	0.922	PM/SMA, M1, subcentral area	4, 6, 43
CH23	1860.4	8453.8	118.8	1627.1	0.326	PM/SMA, M1, S1	3, 4, 6
CH24	795.7	3389.5	185.0	717.6	0.793	PM/SMA, M1, S1	3, 4, 6
CH25	272.0	1032.8	282.3	736.7	0.600	PM/SMA	6
CH26	492.7	1021.9	451.3	1021.9	0.682	PM/SMA, M1, S1	3, 4, 6
CH27	589.0	1872.8	−164.4	3650.2	0.422	PM/SMA, DLPFC	6, 9
CH28	2051.6	5254.1	2216.8	2637.1	0.831	PM/SMA, M1, S1, subcentral area	1, 2, 3, 4, 6, 43
CH29	466.4	1338.3	660.1	1215	0.831	PM/SMA, M1, S1	1, 2, 3, 4, 6
CH30	83.5	574	127.0	441.7	0.781	S1	1, 2, 3
CH31	185.3	585.5	180.2	433.6	0.743	PM/SMA, M1, S1	3, 4, 6
CH32	172.8	683.6	93.7	610	0.658	PM/SMA, M1, S1	3, 4, 6
CH33	395.9	829.5	175.3	1157.8	0.922	PM/SMA, M1, S1	1, 3, 4, 6
CH34	1080.8	1352.9	201.1	3437.5	0.258	PM/SMA, M1, S1	1, 3, 4, 6
CH35	2171.0	3748.9	1716.9	4714.2	0.781	PM/SMA, M1, S1, subcentral area	3, 4, 6, 43
CH36	484.2	2134.7	838.6	1144	0.987	S1, SMGy	1, 2, 3, 40
CH37	126.0	1853.1	258.5	714.4	0.534	S1, SMGy	1, 2, 40
CH38	31.9	2334.7	116.3	1197.5	0.857	M1, S1, SMAss	1, 2, 3, 4, 5
CH39	698.5	1794.4	348.6	1213.2	0.743	M1, S1	1, 2, 3, 4
CH40	594.2	1177.2	174.9	1688.7	0.694	S1, SMGy	1, 2, 3, 40
CH41	734.8	1303.5	337.6	3356.3	0.502	PM/SMA, M1, S1, SMGy	1, 2, 3, 4, 6, 40
CH42	785.4	2923	851.0	1936.2	0.922	STG, SMGy, STGy	22, 40, 42
CH43	406.4	2003.6	673.2	1096	0.793	SMGy	40
CH44	86.2	2509.7	296.0	1147.6	0.844	S1, SMAss, SMGy	2, 5, 40
CH45	553.8	2667.6	77.9	2201.2	0.213	SMAss	5, 7
CH46	440.1	2319.6	245.7	2170.5	0.534	S1, SMAss	2, 3, 5, 7
CH47	1473.9	2606.4	103.6	4190	0.471	S1, SMAss, SMGy	2, 5, 40
CH48	572.7	1265.9	552.7	1665.8	0.368	S1, SMGy	1, 2, 40
CH49	1242.5	3665.2	1589.9	4123.6	0.502	S1, SMAss, SMGy	2, 40, 42
CH50	−46.1	2900.9	336.8	2213.3	0.806	SMGy	40
CH51	533.7	2582.8	754.4	1436.4	0.974	SMGy	40
CH52	304.3	2345.9	125.2	1431.2	0.441	SMAss, SMGy	5, 7, 40
CH53	557.6	1797.2	134.4	1350.6	0.245	SMAss	5, 7
CH54	839.6	2432.6	279.0	3320.7	0.359	SMGy	40
CH55	551.2	1843.3	365.1	1767.6	0.909	SMGy	40

*P*-value; Wilcoxon signed-rank test. *There was a significant difference between the palatable gum and unpalatable gum conditions. Cortical areas corresponding to the channel using the Anatomy 1.8 toolbox for SPM (Eickhoff et al., 2005). Table items show more than 5% of the area. FPC, frontopolar cortex; DLPFC, dorsolateral prefrontal cortex; SEF, supplementary eye fields include frontal eye fields; PM/SMA, pre-motor and supplementary motor cortex; M1, primary motor cortex; S1, primary somatosensory cortex; STG, superior temporal gyrus; SMAss, somatosensory association cortex; SMGy, supramarginal gyrus part of Wernicke’s area; STGy, primary and auditory association cortex; T1, subcentral area (primary taste cortex).

The raw fNIRS data were denoised using the Hemodynamic Modality Separation Method (Yamada et al., 2012). This method was adapted to separate functional and systemic signals based on their hemodynamic differences. This data processing method helped eliminate systemic noise and motion artifacts and extract the hemodynamic changes of the cerebral cortex. A five-point moving average was used to smoothen the waveform, and the amplitude of the fNIRS data was set to zero at the beginning of each gum-chewing trial. We calculated the standard deviation of oxy-Hb 40–30 s prior to chewing (regarded as the baseline) and normalized the data. The statistical analysis was performed on the oxy-Hb data only since the statistical results became essentially the same regardless of the hemoglobin species due to the theoretical assumption in the currently adopted noise-reduction method.

The area under the curve (AUC) was calculated every 30 s to clarify the changes during chewing and was compared to the baseline values. The AUC during the 5 min chewing task was also calculated. Two pairs of prefrontal regions of interest (ROI, Figure 2C) were set to clarify the influence of emotion during palatable and unpalatable gum chewing. The selected regions were the bilateral frontopolar areas (FPC, Brodmann area 10, Hereinafter BA10) and the bilateral dorsolateral prefrontal cortex (DLPFC, BA 9)/supplementary eye field (SEF:BA 8) areas (DLPFC/SEF).

The channel-based AUC values were interpolated on 3,753 voxels (2 mm × 2 mm × 2 mm) on the cortical surface (depth up to 1.8 cm) of the standard MNI brain (Ye et al., 2009; Hirsch et al., 2017; Zhang et al., 2017) to generate an activity map for each condition of each individual. A second-level group analysis was performed using SPM8 software with voxel-wise datasets to compare differences in cortical activity between the two conditions.

### Masseter muscle activity

Electromyograms of the bilateral masseter muscles were measured to monitor orofacial motor output during gum chewing as described previously (Hasegawa et al., 2007). Surface electrodes were attached to the skin over the masseter. Analog signals were amplified using a bio-amplifier (BA-1008, TEAC, Tokyo, Japan) and stored on a personal computer for offline analysis. The sensitivity, time constant, and cut-off frequency of low-pass filter of the amplifier were set to 100 μV, 0.03 sec, and 3 kHz, respectively. Participants were asked to clench their jaws with maximum power for 2 s to calculate the maximum voluntary contraction (MVC). Masseter muscle activity during gum-chewing tests was calculated as the relative MVC value (%).

### Heart rate and autonomic nervous response

Electrocardiography measurements were recorded using a bipolar chest lead. Data were amplified with a biological signal telemeter (Polytele STS; TEAC, Tokyo, Japan) and transferred to a personal computer at a sampling frequency of 1 kHz *via* an A/D conversion card (CBI-3133A; Interface, Hiroshima, Japan). Electrocardiography data were used to obtain heart rate values using a biomedical signal analysis software program (Fluclet^®^; Nagaoka & Co., Ltd., Nishinomiya, Japan). In addition, a fluctuation analysis was conducted to determine the RR interval in electrocardiography using wavelet analysis with a biomedical signal analysis software program. The high-frequency (0.04–0.15 Hz) component of the RR-interval (RR-HF) was calculated as the index of the cardiac vagus nerve activity (Randall et al., 1991). The ratio of low-frequency components to high-frequency (0.15–0.40 Hz) components (RR-LF/HF) was calculated as an index of the cardiac sympathetic nerve activity (Randall et al., 1992).

## Data analysis

Changes in each index were evaluated using the AUC (Hasegawa et al., 2007, 2009, 2013). Using the median values 5 min before gum chewing as the baseline, variations during and after gum chewing were calculated for each index. Changes in each AUC due to the tasks were evaluated using a one-sample Kolmogorov–Smirnov test. In order to assess those regions, the average AUC of the channels included in the two pairs of bilateral ROIs were calculated, and two-way ANOVA with the main effects of the ROI (FPC or DLPFC/SMA) and condition (palatable or unpalatable) and the first-order interaction effect between them were analyzed. The multiple comparisons with Wilcoxon signed-rank test (*p*-values were Bonferroni-adjusted) were applied to determine whether there was a statistically significant difference in the oxy-Hb change in each ROI during palatable and unpalatable gum chewing (*p* < 0.05).

To evaluate temporal changes, representative values were calculated every 30 s. A repeated analysis of variance was performed to investigate changes in each index between baseline (median value for 5 min before chewing started) and each 30-s interval after chewing. If a difference was significant, comparisons were made between values before gum chewing and those at other intervals using Dunnett’s test. To compare two samples (palatable gum vs. unpalatable gum and left vs. right for the same position channel), the paired *t*-tests (normal distributions) or Wilcoxon signed-rank tests (non-normal distributions) were performed. Pearson correlation coefficient (normal distributions) or Spearman’s rank correlation (non-normal distributions) was used to analyze the correlation between the AUC of oxy-Hb and the sensory evaluation.

All statistical analyses were performed using a commercially available software package (IBM SPSS Statistics, version 22.0.0 for Windows; SPSS, Armonk, NY, USA). The level of significance was set at 5%.

## Results

### Sensory test

**FIGURE 3:**
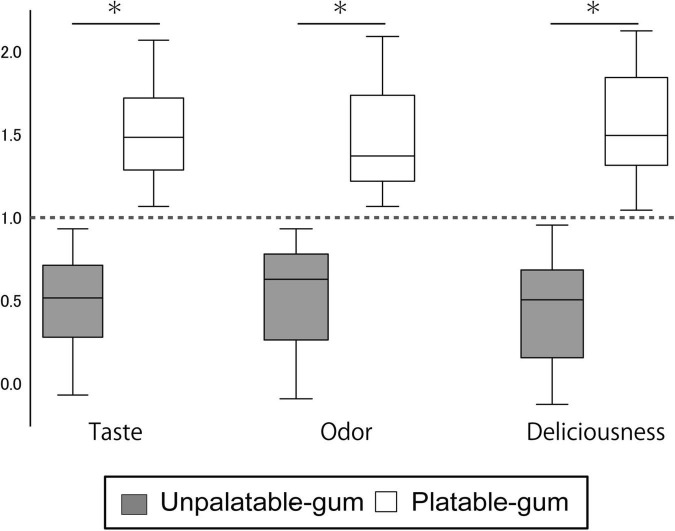
Results of sensory testing. The Y-axis scale shows the sensory testing results. Data for each gum were calculated using the following formula. Palatable gum = X/{(X + Y)/2}, Unpalatable gum = Y/{(X + Y)/2}; where X: VAS of palatable gum, Y: VAS of unpalatable gum. *Indicates a significant difference between unpalatable gum and palatable gum (paired *t*-test, *P* < 0.001).

The estimates of the taste, odor, and deliciousness of the gums by the participants are shown in Figure 3. From the 36 subjects, one female evaluated the unpalatable gum to be better than the palatable gum in “taste” and “deliciousness.” This subject was excluded from the evaluation for the sensory test results of the two gums that reflect palatable/unpalatable emotion. The subjective ratings significantly differed between the two gum types.

**FIGURE 4:**
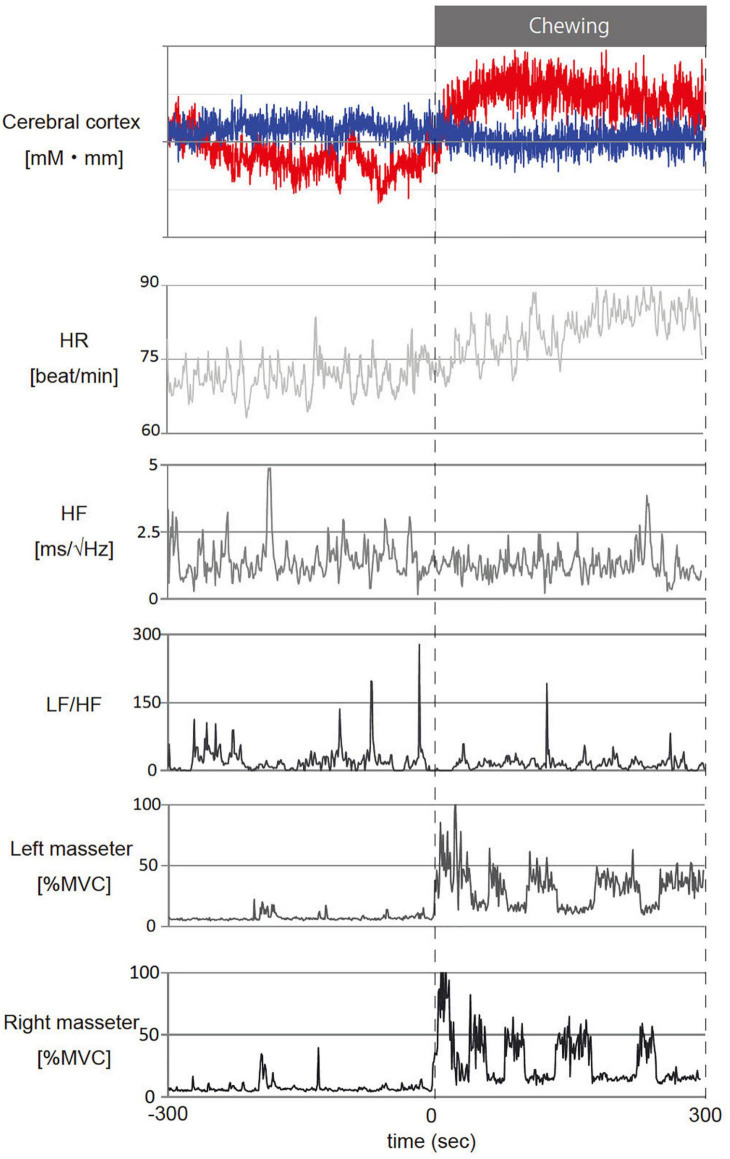
Representative temporal changes in oxy- and deoxy- hemoglobin (Hb) in channel 24, heart rate (HR), cardiac vagus nerve activity (RR-HF), cardiac sympathetic nerve activity (RR-LF/HF) and left/right masseter mass activity (l-%MVC, r-%MVC) during gum chewing. The red line in the cerebral cortex shows oxy-Hb and the blue line shows deoxy-Hb. –300 to 0 s: Before chewing and 0 to 300 s: During chewing. The calculations represent the values per second for the data.

Figure 4 shows representative fNIRS data (corresponds to channel 24), heart rate, cardiac vagus nerve activity (RR-HF), cardiac sympathetic nerve activity (RR-LF/HF), and left/right masseter mass activities during pre- and post-chewing.

### The effect of chewing on hemodynamic responses

**FIGURE 5:**
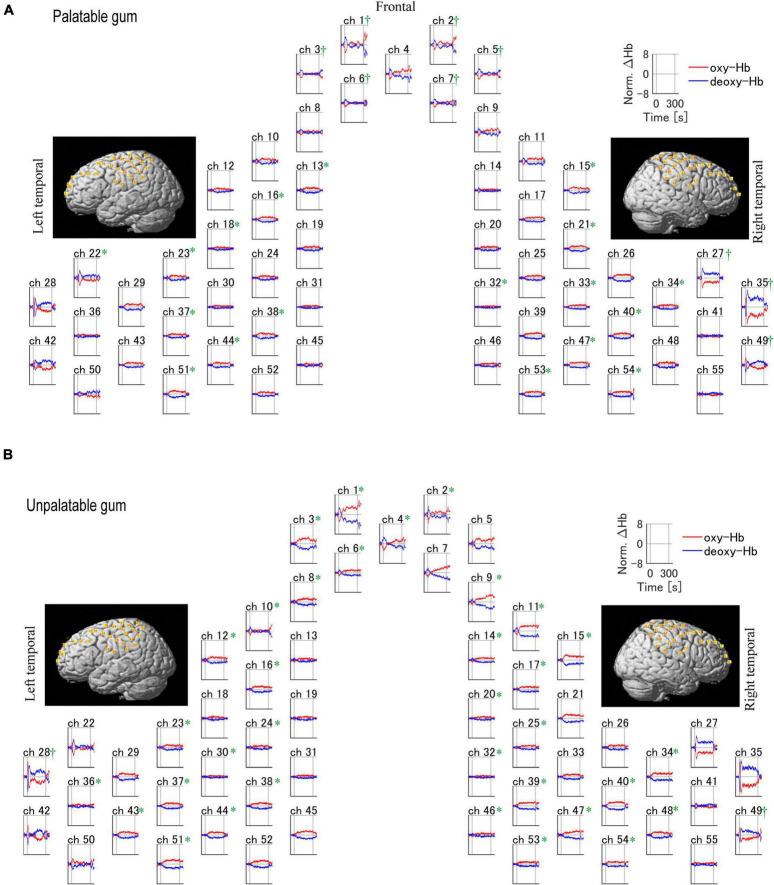
Temporal profiles of changes of the hemodynamic response during the chewing of palatable/unpalatable gums. The average waveform for all participants is shown. **(A)** Palatable gum; **(B)** unpalatable gum. *A significant increase in the area under the curve (AUC) was observed after chewing relative to baseline (*p* < 0.05). ^†^A significant decrease in AUC was observed after chewing relative to baseline (*p* < 0.05). The one-sample Kolmogorov–Smirnov test.

Figure 5 shows the temporal changes in oxy- and deoxy-Hb levels for each of the 55 channels. Oxy-Hb levels increased in the primary motor cortex (palatable gum: ch22, 23, 32–34, 38, unpalatable: ch23–25, 28, 32, 34, 38, 39) and primary somatosensory cortex (palatable gum: ch32–34, 37, 38, 40, 44, 47. unpalatable: ch23–25, 30, 32, 34, 37–40, 46–48) during gum chewing regardless of the type of gum. This area is known as the cortical masticatory area (Lund et al., 1984; Hatanaka et al., 2005). The channels in the prefrontal cortex, which correspond to the BA 10, showed decreased oxy-Hb during gum chewing. Ch1–3 showed a decrease in oxy-Hb in both gum chewing conditions. Oxy-Hb decreased in the frontal cortex only in ch4 during unpalatable gum chewing, but in ch5–7 during palatable gum chewing, a wide range of decreases in oxy-Hb were shown. The temporal changes in oxy-Hb in these channels showed a decrease immediately after the start of chewing and then increased after 30 s. Chewing unpalatable gum resulted in an increase in oxy-Hb levels and a significant increase in AUC in some channels (ch6, 8, 9) located at BA8–10 than before chewing. This increase, however, was not observed with palatable gums. Ch27, 35, and 49 (BA3, 4, 6, 9, and 40, 42, 43 in the right hemisphere) showed a significant decrease in the AUC during palatable gum chewing, while Ch28 and 49 showed a significant decrease in the AUC during unpalatable gum chewing.

### Comparison of the hemodynamic responses between palatable and unpalatable gum

**FIGURE 6:**
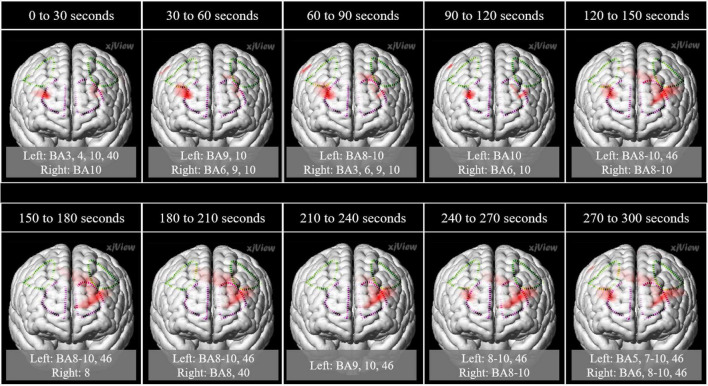
Comparison of the hemodynamic response during the chewing of the two gums. Parametric mapping analysis (paired *t*-test; uncorrected *p* < 0.05) was performed using the area under the curves (AUCs) (palatable gum vs. unpalatable gum). Areas with significant differences in Unpalatable > Palatable are shown. 3D image was obtained from images obtained every 30 s using MRIcroGL (https://www.mccauslandcenter.sc.edu/mricrogl/). Regions of interest (ROI) in the prefrontal cortex are circled by dotted lines. Purple dotted line: Frontopolar cortex, green dotted line: Dorsolateral prefrontal cortex and includes frontal eye fields.

In the left frontal pole cortex (channel 3), there was a statistically significant increase in oxy-Hb levels during the unpalatable gum chewing than during the palatable gum chewing (Table 1). The left frontopolar and DLPFC regions (channels 1, 3, 6, and 8) showed a similar trend, wherein oxy-Hb levels increased more with the chewing of unpalatable gum than with palatable gum. Figure 6 shows the difference in AUC of oxy-Hb between palatable and unpalatable gum chewing every 30 s. In the bilateral frontal cortex area (corresponding to the frontopolar and DLPFC/SEF, Figure 6), after 0–150 s from the start of gum chewing, the oxy-Hb value during chewing of unpalatable gum was higher than during palatable gum chewing. In the left frontal area, all 30-s segments after the start of gum chewing were also higher than that during chewing palatable gum. From 150 to 300 s after the start of chewing, the oxy-Hb value during chewing unpalatable gum was higher in the left frontal region than during palatable gum chewing.

The AUCs of oxy-Hb during gum chewing were also compared between the hemispheres. The pre-motor and supplementary motor cortex/supplementary eye fields, including the frontal eye field cortex, of the left hemisphere, showed higher values than those of the right hemisphere while chewing both palatable and unpalatable gums. Although no significant difference was found between the two gums during the 5 min of chewing, the comparisons made between the gums in segments of 30 s each showed that oxy-Hb during unpalatable gum chewing was often higher than that of palatable gum chewing. This suggests that the hemodynamic response was stronger for unpalatable gum than for palatable gum.

**FIGURE 7:**
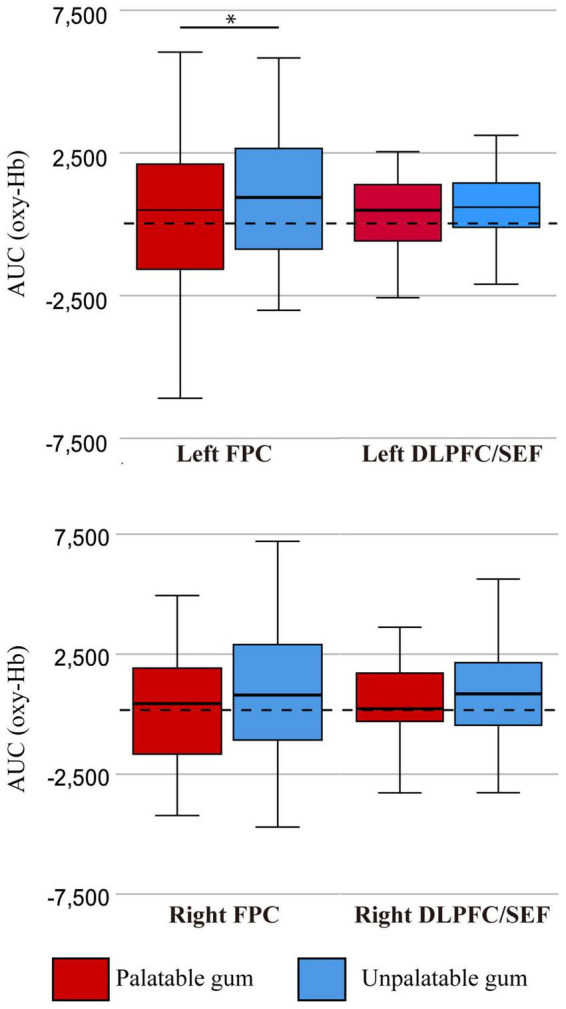
The oxy-Hb changes per region of interest (ROI). FPC, frontopolar cortex, DLPFC/SEF, dorsolateral prefrontal cortex and includes frontal eye fields. AUC (oxy-Hb), mean area under the curve (AUC) of oxy-hemoglobin of the channels in each regions of interest. *There was a statistically significant difference between the palatable gum and the palatable gum by multiple comparison using Wilcoxon signed-rank test (*p*-values were Bonferroni-adjusted).

Figure 7 shows the comparison of oxy-Hb changes between palatable and unpalatable gum chewing. The result of two-way ANOVA left AUC of oxy Hb was significantly higher in unpalatable gum chewing than in palatable gum chewing [F(1,68) = 4.42, *p* = 0.039, partial η2 = 0.061]. On the other hand, there was no significant difference in the AUC of oxy Hb among the different areas of ROI [F(1,68) = 0.031, *p* = 0.860, partial η2 = 0.0005], and no significant difference in the interaction [F(1,68) = 3.60, *p* = 0.062, partial η2 = 0.05]. The difference in oxy-Hb change between the gums was only significant in the left FPC, although there was a trend toward greater oxy-Hb change for unpalatable gums than palatable gums (Figure 4).

### The correlations between subjective evaluations and hemodynamic responses

Table 2 shows a significant correlation between each channel’s hemodynamic response and the sensory test results. In the left frontal channel 8, which corresponds to the DLPFC and supplementary eye field (SEF), the cerebral hemodynamic response was weak and negatively correlated with the sensory test result.

**TABLE 2:** The correlations between subjective evaluations and hemodynamic responses.

	RTaste	*P-value*	ROdor	*P-value*	RDeliciousness	*P-value*	Applicable cortical region
ch8	–0.258	0.03	–	–	−0.272	0.023	DLPFC, SEF

The table displays only the channels that showed a significant correlation between the sensory test results and oxy-Hb. DLPFC, dorsolateral prefrontal cortex; SEF, supplementary eye fields include frontal eye fields.

### Heart rate, autonomic nerve activity, and masseter muscle activity

**FIGURE 8:**
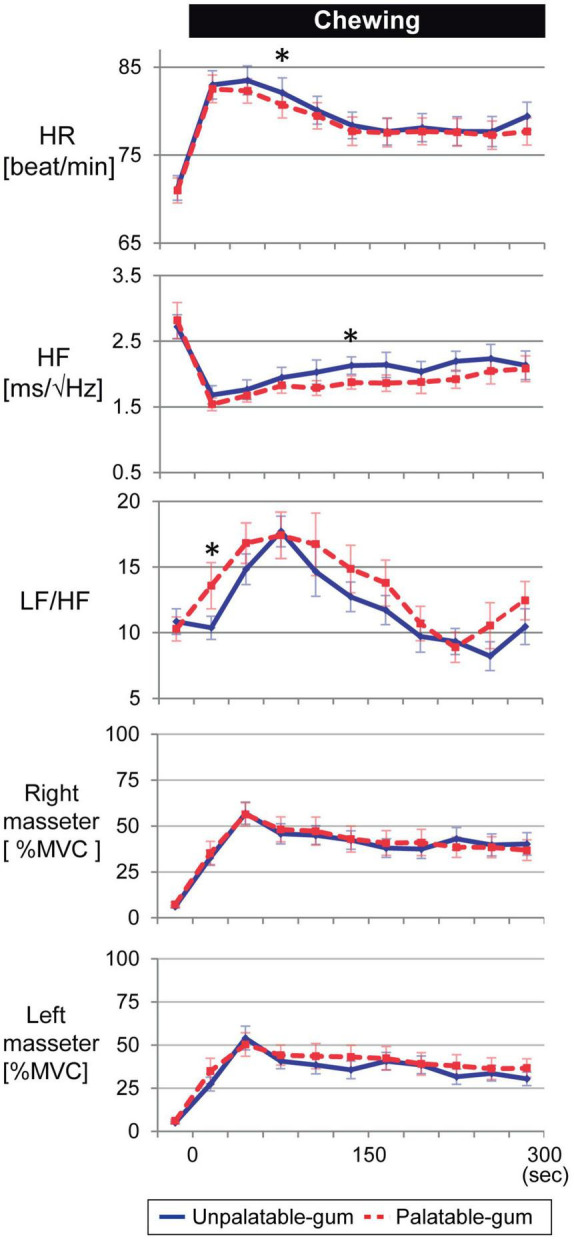
Temporal changes in heart rate, autonomic nerve activity, and bilateral masseter muscle activity for every 30 s of gum chewing. *Indicates a significant difference between palatable and unpalatable gums. Blue line: unpalatable gum. Red line: palatable gum. A repeated analysis of variance was performed to investigate changes in each index between baseline (median value for 5 min before chewing started) and each 30-s interval after the start of chewing. If a difference was significant, comparisons were made between values before gum chewing and those at other intervals using Dunnett’s test.

Figure 8 shows that the heart rate significantly increased while chewing both palatable and unpalatable gums. No significant difference in heart rate was observed in 5 min of chewing duration between the types of gums. The parameters were also compared between the types of gums every 30 s. In the beginning, 60–90 s after the start of chewing, the heart rate corresponding to unpalatable gum was higher than that of palatable gum. Decreased HF and increased LF/HF were significant during the chewing of both types of gums. These parameters showed significant changes immediately after the start of chewing. No significant differences were observed in the AUCs of either HF or LF/HF while chewing each type of gum. On the other hand, significant differences were observed in heart rate and autonomic nerve activity at a certain 30-s duration between the gums. When masseter muscle activity was compared between the types of gums, no significant difference was observed. Thus, there were no differences between the two types of gums in terms of cardiovascular or muscle activity, and the momentum for each type of gum was almost identical; differences in emotion caused no differences in momentum.

## Discussion

This study was conducted to understand the cortical hemodynamic responses related to chewing of gums with different tastes/odors using the fNIRS system. Hemodynamic responses were significantly increased in the bilateral primary sensorimotor cortex during gum chewing compared to the resting conditions. In addition, unpalatable gum chewing showed an increased hemodynamic response in the left frontal channel. There were significant differences in the hemodynamic responses in BA10, corresponding to the frontopolar cortex, between the palatable and unpalatable gum types during the 5 min of chewing.

It is known that the anterior insula and adjoining frontal opercula cortex constitute the primary taste cortex, and the caudal parts of the orbitofrontal cortex constitute the secondary taste cortex (Ogawa et al., 1989; Baylis et al., 1995). These two cortical areas have been shown to be activated by taste in human neuroimaging studies using positron emission tomography (PET) and f-MRI (Small et al., 2001; Del Parigi et al., 2002; Zald et al., 2002; Kringelbach et al., 2004; Small and Prescott, 2005; Mihara et al., 2008). We previously evaluated the effects of taste and flavor on blood flow changes in the frontal cortex (Hasegawa et al., 2013). In this study, we evaluated the grand and time-binned averages of oxy-Hb concentration changes to compare cortical activity during chewing palatable and unpalatable food. The former was employed to evaluate overall activity changes with the chewing experience with pleasant and unpleasant emotions, and the latter was to determine the transient changes in the cortical activity during chewing a flavored gum, whose intensity of taste and odor stimuli lose over time. There was a trend toward greater oxy-Hb change for the unpalatable gum than for the palatable gum, and the difference in oxy-Hb change between gums was significant only in the left FPC among all ROIs. In the frontopolar and DLPFC/SEF, after 0–150 s from the start of gum chewing, the oxy-Hb value during chewing of unpalatable gum was higher than during palatable gum chewing.

We found that when participants chewed palatable gum, hemodynamic responses were significantly higher than those obtained when they chewed gum without any taste and odor or just sweet, even though the masseter muscle activity was not significantly different among the three gum chews. No significant hemodynamic changes were observed when the same taste and flavor samples as those of the palatable gum were placed in the mouth. The results suggest that taste and odor influence brain activity during mastication in sensory, cognitive, and motivational processes, rather than in the motor control (Hasegawa et al., 2013). Therefore, in the present study, only palatable and unpalatable gum chewing was performed, not tasteless and odorless gum chewing, which served as a control.

Palatable gum has been marketed for many years in Japan and is known to have a popular taste and odor; thus, the majority of participants might be able to chew this gum with minimal attention, such as during semi-voluntary exercise (e.g., walking) for 300 s. Conversely, unpalatable gum has a displeasing flavor and is not delicious for most Japanese individuals. Moreover, the participants were under stressful/negative-emotion conditions when they were asked to chew according to the metronome’s rhythm. Thus, the participants had to exert greater effort with respect to the act of chewing in comparison to that of palatable gum. As a result, chewing unpalatable gum was an exercise requiring more voluntary and intentional awareness than chewing palatable gum. Cortical activity in the left frontopolar cortex and DLPFC, which we defined as ROIs in this study, showed a higher value when chewing unpalatable gum than palatable gum.

In this study, cortical activity in the left prefrontal cortex, corresponding to the frontopolar cortex, DLPFC, and supplementary eye fields (BA 8, 9, 10, 46, 44, 45, lateral 47) increased during unpalatable gum chewing compared to that during palatable gum chewing. BA 4, 6, 9, 8 collect sensory information from all modalities undergoing higher-order processing *via* bidirectional nerve fiber connections between the temporal and parietal association areas. At the same time, BA10 receives inputs of emotional and arousal states through nerve fiber connections with the limbic system, including the hippocampus, cingulate cortex, and amygdala, as well as with the brainstem (Ono et al., 2015a,b). BA10 is also known to be the highest-order cognitive processing area that integrates and controls information from other prefrontal cortex areas. The prefrontal cortex (the frontal pole is part of the prefrontal cortex) and the striatum are involved in the reward prediction process, and anatomically the prefrontal cortex and striatum are closely connected (Ono et al., 2015a,b). Pan et al. (2014) suggested that striatal neurons may only be able to predict reward for novel stimuli. In the present study, it is possible that the striatal neurons were excited by the novel stimuli because the taste of the unpalatable gum was unknown, resulting in increased FPC activity, whereas the known and learned palatable gum did not excite the striatal neurons.

The prefrontal cortex, responsible for appetite control, is thought to be the center of cognitive functions such as attention, learning, thought, memory, and action. In addition, the DLPFC is a critical brain area associated with appetitive control, food craving, and executive functioning, and controls the executive function, which is deeply related to memory, attention, learning, and behavior (Goldman-Rakic, 1996; Miller, 1999; Rowe et al., 2000); PET studies have found that changes in the DLPFC are related to reward value, and regulation of limbic reward regions (Small et al., 2001; Del Parigi et al., 2002). Kringelbach et al. (2004) showed that activation of the left DLPFC region was associated with taste or taste-odor combinations (Gluck et al., 2017). The prefrontal cortex, including the DLPFC, is also known to be responsive to exercise (Colcombe et al., 2006). During low-intensity exercise, the left DLPFC activity is evoked (Byun et al., 2014). In addition, young adults typically demonstrate left-lateralized frontal activation during light-intensity exercise (Hyodo et al., 2016). Notably, chewing movement is a light-intensity exercise; therefore, our results are consistent with those of previous studies. It has been reported that occlusal discomfort, an unpleasant emotion of oral movement origin, increased hemodynamic response in the frontopolar cortex and DLPFC (Ono et al., 2015a), and the present results also implicate the DLPFC in the regulation of emotion. Lorenz et al. reported that DLPFC activity was negatively correlated with perceived intensity and unpleasant emotion (Lorenz et al., 2003), and Minematsu et al. (2018) showed that hedonically positive stimuli decreased oxy-Hb and hedonically negative stimuli increased oxy-Hb in the anterior prefrontal cortex, which is consistent with our results. The valence-lateralization theory, f-MRI studies in healthy subjects demonstrated linear or parametric dependence of negative and positive emotional judgments on neural activity in left and right DLPFC (Grimm et al., 2006). Grimm et al. (2008) reported that healthy subjects showed a negative correlation between left DLPFC neural activity and positive emotional valence by f-MRI study. This report is consistent with our results and may reflect the processing of emotions by chewing at DLPFC. Our result supposed that unpleasurable negative valence may cause greater cortical activity in the frontopolar cortex and DLPFC than pleasant positive valence.

For oxy-Hb changes in the frontal lobe, the left hemisphere level was significantly increased by chewing compared to that in the right hemisphere, possibly because of the presence of a language center in the left hemisphere (Paulesu et al., 1993). Since all the participants in this study were right-handed, the language center was likely located in the left hemisphere. Cortical activity changes in the primary sensorimotor cortex were increased compared to those in the frontal lobe, likely because chewing is considered a semi-voluntary exercise. In the frontal association area, the anterior portion was responsible for higher information processing. For example, the lateral frontal association cortex plays an important role in complicated, abstract cognitive manipulation. In addition, cortical activity in the bilateral hemispheres was the highest approximately 90 s after the start of chewing, and a tendency to maintain such a peak during chewing was also shown in our previous study (Hasegawa et al., 2007). Notably, gum chewing increases oxy-Hb because it is a low-intensity exercise (Hasegawa et al., 2007, 2013). Aoki et al. showed that the prefrontal cortex plays a role in the interaction between mood and cognition in everyday situations (Aoki et al., 2011).

Our results show that blood circulation and chewing muscle activity were comparable between the two gum types. We found weak negative correlations between subjective evaluations and cortical activity changes in the left frontal cortex. Cortical activity increases with brain activity, and right hemisphere superiority is likely related to the stimulation of the taste (Small et al., 1999) and odor (Zatorre et al., 1992; Small et al., 1997)pathways. Stimulation with palatable gum (a familiar flavor for participants) might have caused reduced activation of the left prefrontal cortex. In addition, since the SEF (the anatomical area of the dorsal medial frontal lobe) receives input from DLPFC, and the SEF neurons are known to carry signals related to response evaluation, it is possible that the neural activity in the area was increased during the series of chewing the unfamiliar flavor gum, which elicited negative feelings of not palatable (Stuphorn, 2015).

The present study has some limitations. It is important to note that there is no conclusive evidence to support the hypothesis that emotion or preference is asymmetrically represented in the brain area. Because fNIRS monitors changes in cortical activity on the surface of the cortex, the activity of the areas deeply involved in taste/odor (i.e., gustatory and olfactory neural circuits) cannot be evaluated in this experiment. In the orbital frontal cortex, BA10 was evaluated by many channels, but the cortex corresponding to BA13 and 14 was not covered in this study. To evaluate emotional changes during eating, channel placement targeting the orbitofrontal cortex should be considered in the future. Since Sato et al. (2011) reported that salivary secretion (hemodynamic changes in the arteries around the parotid gland) in response to taste stimuli correlates well with the DLPFC, we should have considered whether there is a difference in salivation between the two gums.

## Conclusion

Regardless of gum type, the cortical hemodynamic response was increased in the primary sensory-motor cortex area in both hemispheres during chewing. Emotional changes due to taste and odor modulated the left frontopolar cortex and dorsolateral prefrontal cortex during gum chewing. However, the relationships between such emotional changes and cerebral cortex activity could not be represented by a simple correlation. The palatable/unpalatable emotion during chewing modulated activity in the frontal pole and prefrontal cortex, suggested that the hemodynamic response to chewing with unpleasant emotion was similar to the hemodynamic response that occurs during unpleasant stimuli.

## Data availability statement

The raw data supporting the conclusions of this article will be made available upon request to the corresponding author YH, cem17150@dent.niigata-u.ac.jp, pending a formal data sharing agreement and approval from the Local Ethics Committee.

## Ethics statement

The studies involving human participants were reviewed and approved by the Ethics Committee of the Hyogo College of Medicine (approve num-2209). The patients/participants provided their written informed consent to participate in this study.

## Author contributions

YH, AS, TS, JS, MS, YO, and TO conceived and designed the research and reviewed the data. YT and HK critically revised the manuscript. YH, AS, TS, JS, and YO collected the data, performed the statistical analysis, and drafted the manuscript. All authors have reviewed the manuscript.
